# Experimental study of the effects of hypoxia simulator on osteointegration of titanium prosthesis in osteoporotic rats

**DOI:** 10.1186/s12891-021-04777-6

**Published:** 2021-11-11

**Authors:** Jiangfeng Liu, Huijun Kang, Jiangfeng Lu, Yike Dai, Fei Wang

**Affiliations:** Department of Joint Surgery, Third Hospital of Hebei Medical University, Ziqiang Road 139, Shijiazhuang, 050051 China

**Keywords:** Deferoxamine, Osseointegration, Osteoporosis, Arthroplasty, Titanium prosthesis

## Abstract

**Background:**

Poor osseointegration is the key reason for implant failure after arthroplasty,whether under osteoporotic or normal bone conditions. To date, osseointegration remains a major challenge. Recent studies have shown that deferoxamine (DFO) can accelerate osteogenesis by activating the hypoxia signaling pathway. The purpose of this study was to test the following hypothesis: after knee replacement, intra-articular injection of DFO will promote osteogenesis and osseointegration with a 3D printed titanium prosthesis in the bones of osteoporotic rats.

**Materials and methods:**

Ninety female Sprague–Dawley rats were used for the experiment. Ten rats were used to confirm the successful establishment of the osteoporosis model: five rats in the sham operation group and five rats in the ovariectomy group. After ovariectomy and knee arthroplasty were performed, the remaining 80 rats were randomly divided into DFO and control groups (*n* = 40 per group). The two groups were treated by intraarticular injection of DFO and saline respectively. After 2 weeks, polymerase chain reaction (PCR) and immunohistochemistry were used to evaluate the levels of HIF-1a, VEGF, and CD31. HIF-1a and VEGF have been shown to promote angiogenesis and bone regeneration, and CD31 is an important marker of angiogenesis. After 12 weeks, the specimens were examined by micro-computed tomography (micro-CT), biomechanics, and histopathology to evaluate osteogenesis and osseointegration.

**Results:**

The results of PCR showed that the mRNA levels of VEGF and CD31 in the DFO group were significantly higher than those in the control group. The immunohistochemistry results indicated that positive cell expression of HIF-1a, VEGF, and CD31 in the DFO group was also higher. Compared with the control group, the micro-CT parameters of BMD, BV/TV, TB. N, and TB. Th were significantly higher. The maximal pull-out force and the bone-to-implant contact value were also higher.

**Conclusions:**

The local administration of DFO, which is used to activate the HIF-1a signaling pathway, can promote osteogenesis and osseointegration with a prosthesis in osteoporotic bone.

## Background

Implant stability and the long-term success of total knee arthroplasty depend on component fixation that can either be cemented or cementless [1, 2]. Prosthesis stability depends not only on good initial mechanical stability, but also on late osseointegration, whether in normal or osteoporotic bone. However, most patients undergoing arthroplasty are older people who have varying degrees of osteoporosis. Osteoporosis is characterized by bone resorption exceeding bone formation, low bone mass, and microstructural degeneration [3]. Recent studies have indicated that low bone mass around prostheses leads to initial instability, aseptic loosening, periprosthetic fracture, and an increased rate of revisions. Moreover, relevant problems associated with low bone mass around prostheses in osteoporosis patients are more obvious [4–7]. Therefore, it is necessary to take measures to increase the amount of bone around the prosthesis and improve the rate of osseointegration to prevent loosening of the prosthesis loosening. Additionally, biological enhancement of cancellous bone quantity and osseointegration will provide one mechanism to improve the outcomes of cementless total knee arthroplasty. Unfortunately, there is still no good solution for protecting bone mass around prostheses. Currently, in addition to improvement of the prosthesis surface properties (such as titanium spraying of the surface, a porous titanium trabecular surface, and hydroxyapatite coating) and improved surgical techniques, many drugs have been used in experiments to strengthen bone formation around the prosthesis and increase osseointegration [8–12].

Studies have shown that bone regeneration can be promoted by activating the hypoxia inducible factor-1a (HIF-1a) signal pathway through the use of hypoxia-mimicking drugs or related genetic manipulation. HIF-1a, as a response element, can stimulate the production of vascular endothelial growth factor (VEGF) when combined with the target gene, and finally promote new bone formation through the angiogenic–osteogenic coupling pathway [13–15]. Deferoxamine (DFO) is an iron chelatorthat has recently been widely studied as a hypoxia simulator [16–20]. Ferric iron is a critical cofactor for the prolyl hydroxylase proteins involved in the oxygen-dependent regulation of HIF-1a, and chelation results in the unrestricted activation of HIF-1a. Researchers have shown that local administration of DFO can promote the formation of new bone;this has been confirmed in fracture, distraction osteogenesis, and bone defect animal models [16–21]. Some experiments have shown that DFO can promote bone formation under abnormal bone conditions such as osteoporosis or pathological fracture caused by radiation [22–24]. Jia et al. [25] reported that DFO enhanced the healing of osteoporotic bone defects via enhanced angiogenesis and osteogenesis. However, it is unclear whether DFO can promote the osseointegration of titanium prostheses in osteoporotic bone after knee replacement. In this study, we aimed to test the hypothesis that intra-articular injection of DFO can promote osteogenesis and osseointegration with a 3D printed titanium prosthesis in the bones of osteoporotic rats.

Although the blood supply of cancellous bone is abundant in osteoporotic bone, the expression of HIF-1a and VEGF is lower in osteoporotic bone than in normal bone. The number of H-type blood vessels decreases in osteoporotic bone, followed by a decline in neovascularization and a gradual decrease in local osteogenesis [26]. The loss of bone mass ultimately leads to increased fracture incidence and a higher rate of loosening of the prosthesis after arthroplasty. It is possible to increase new bone mass by using drugs to promote neovascularization around implants after prosthesis implantation. The aim of current study was to use a novel implant model in osteoporotic rats to determine whether local administration of DFO to activate the HIF-1a signaling pathway can augment the cancellous bone mass around the implant and enhance osteointegration.

## Materials and methods

All the animals used were from the Experimental Animal Center of Hebei Medical University. All procedures involving animals are in compliance with the “Animal Care and Use” guidelines and approved by the Animal Care and Experimental Committee of the Third Affiliated Hospital of Hebei Medical University. The ethics approval ID is Z2018–005-1.

### Prosthesis design

**Fig. 1:**
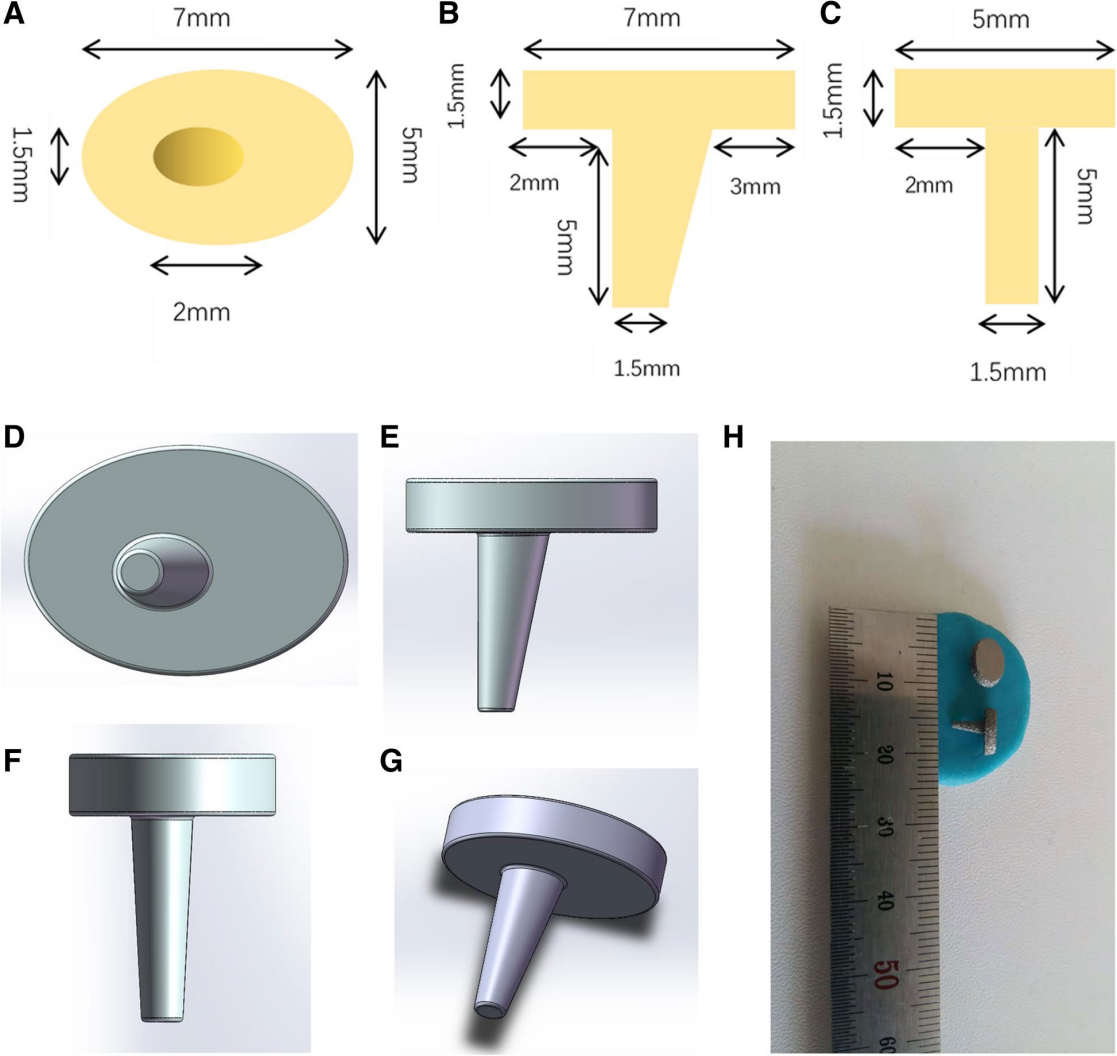
Measurement of the rat tibial prosthesis in transverse (**A**), coronal (**B**), and sagittal sections (**C**); 3D composite image (**D–G**) and entity image (**H**)

For the fabrication of a 3D printed tibial knee prosthesis in rats, the shape and size of the upper tibia, including the medullary cavity, were observed and measured on the transverse, coronal and sagittal planes by dissecting the upper tibia of the rats. Then, a tibial knee replacement (hemiarthroplasty) was designed to replace the tibial articular surface. Based on the implant shape used in human knee replacement, a press-fit titanium tibial implant comprising an articular baseplate and intramedullary stem was fabricated. The baseplate surface was oval in shape with a medial–lateral width of 7.0 mm, anterior–posterior depth of 5.0 mm, and thickness of 1.5 mm. The area of the baseplate was similar to that of the rat tibial plateau, whichforms the joint with the femoral condyle. The stem was approximately columnar, and was 5.0 mm in length and 1.5 mm in diameter. To make the prosthesis stable, a small wing was attached to one side of the stem, which not only prevented the prosthesis from rotating in the bone, but also better adapted to the shape of the medullary cavity of the upper tibia of rats. A 3D image of the prosthesis was simulated by computer-aided design, and the prosthesis was then fabricated with a 3D powder printer (Arcam Q10, Sweden) with titanium powder. The powder particles were spherical in shape with a median size of approximately 40 μm (Fig. 1). The articular surface was polished using 1200 grit silicon carbide grinding paper, and the stem surface was coated with close-packed 40-μm-diameter titanium spheres, providing a mean surface roughness of 12.65 um that was conducive to bone ongrowth. The yield strength of the prosthesis stem was 840.29 Mpa. Implants were degreased and cleaned with Alconox detergent (Seebio, Xi’an, China) and sonication in deionized water. Before implantation, the grafts were sterilized in a standard autoclave (121 °C; humidity 100%; 30 min).

### Establishment of osteoporosis rat model

Ninety 2-month-old female Sprague–Dawley rats were obtained from the Laboratory Animal Center of Hebei Medical University (Shijiazhuang, China) and housed in the Research Animal Facility. The facility is controlled for temperature, ventilation, and illumination. Rats were fed standard hard chow and water ad libitum. When the rats reached the age of 3 months, five rats were randomly designated as members of the sham group. The remaining rats were subjected to ovariectomies (OVX) to simulate osteoporosis. Preoperative subcutaneous injections of gentamicin (5 mg/kg) were administered to protect the rats from infection. Anesthesia was successfully achieved by intraperitoneal injection of 1.5% phenobarbital sodium 40 mg/kg (Schering-Plough, Belgium). One small incision (1.5 cm) was made through the skin and the muscle wall on each side of the backbone on the dorsal aspect, and the bilateral ovaries were exposed and removed from OVX animals. In the sham animals, only a small amount of fat around the ovaries was removed. The wound was sutured in layers. After 3 months, five sham rats and five OVX rats were sacrificed, and the fifth lumbar vertebrae were removed. The bone mineral density (BMD) of the fifth lumbar vertebra of each rat was measured using dual-energy X-ray absorptiometry (DEXA; Lunar Corporation, USA) on the spot. A reduction in BMD of more than 20% was considered to be a successful model of osteoporosis [27, 28].

### Prosthesis implantation surgery

**Fig. 2:**
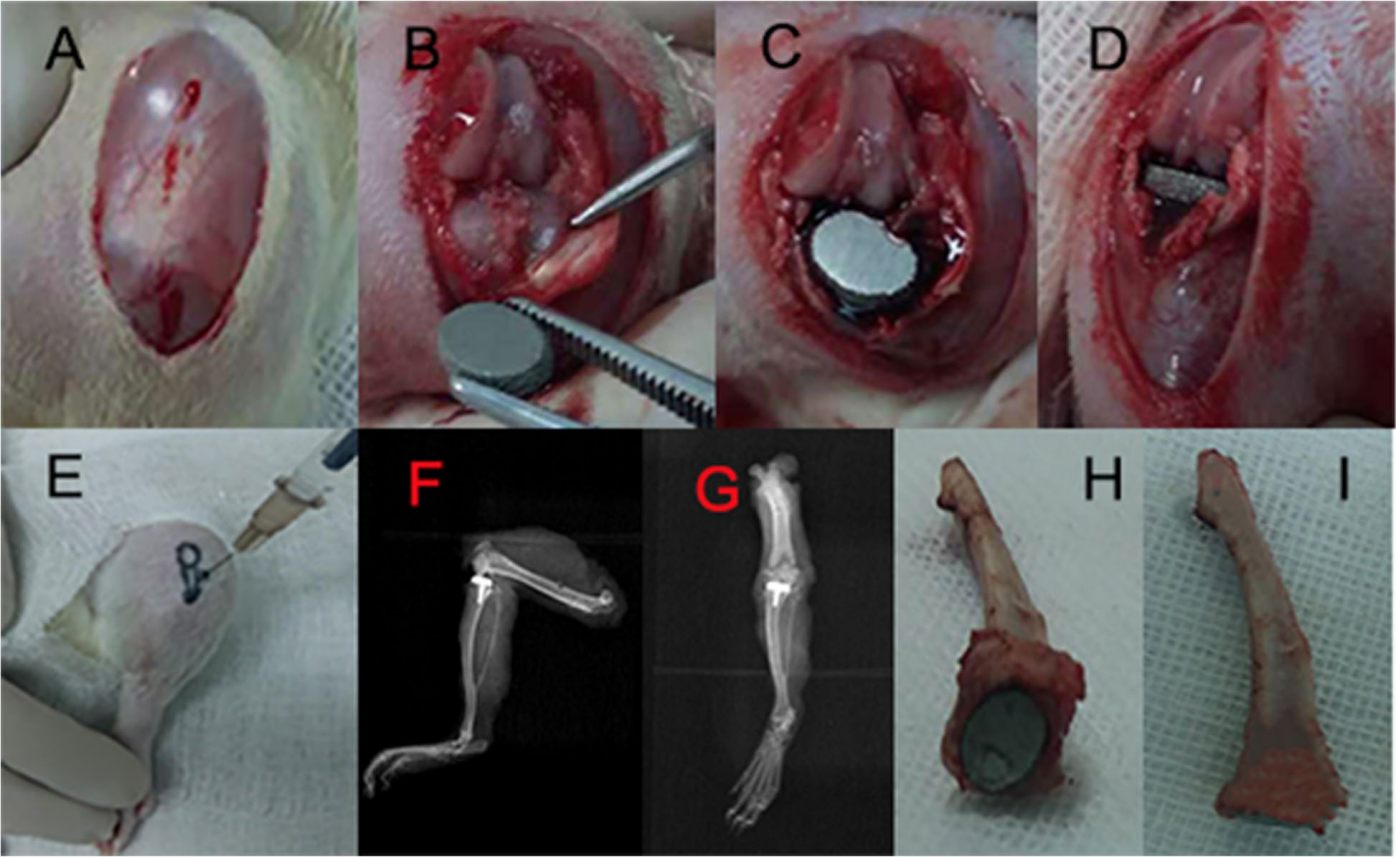
**A–D** The tibial plateau cartilage was replaced by a 3D printed prosthesis through hemiarthroplasty of the knee. **E** Intra-articular injection of deferoxamine (DFO) via a medial approach beside the patellar ligament. **F–G** Postoperative X-ray examination showing that the implant is well positioned. **H–I** Gross tibial specimen with the prosthesis

After confirming osteoporosis in the rats, a unilateral (left) tibial knee replacement was performed on each animal(80 rats). The skin over the left knee was sterilized twice with alcohol. The fur was removed with a hair razor. Preoperative subcutaneous injections of gentamicin (5 mg/kg) were administered to prevent infection, and preoperative subcutaneous injections of buprenorphine (0.03 mg/kg) were administered to relieve pain. After successful anesthesia according to the above methods, the operation area was covered with a sterile sheet leaving only the knee joint exposed. The knee was opened with a parapatellar medial incision and the tendon with the patella was dislocated laterally. After the joint cavity was completely exposed, the anterior cruciate ligament and menisci were resected, and the posterior cruciate ligament was preserved. Bone scissors were used to remove the articular cartilage and proximal epiphysis to accommodate the implant; that is, the entire tibial plateau was removed. The thickness of the cut bone was approximately 1.5 mm, which is equivalent to thickness of the prosthesis tray. A hand drill was used to drill a 1-mm-wide and 5-mm-deep hole in the tibia to fit the stem of the prosthesis. After washing the bone surface with aseptic water, the prosthesis was implanted in a press-fit manner (cementless). Ethibond 4–0 sutures were used to close the joint capsule and Vicryl 5–0 suture was used to close the skin (Fig. 2). After the operation, the knee joint was stable and could be extended and flexed as normal.

### Delivery of DFO

Eighty rats were randomly divided into the DFO group (*n* = 40) and the control group (*n* = 40). Starting on postoperative day 4, all rats in the DFO group were injected with 200 uL of 200 umol DFO solution (Sigma, St. Louis, MO, USA) in normal saline directly into the knee joint cavities. The rats were mildly sedated before the injection, and a 25-gauge needle was used to carefully deliver the solution (Fig. 2). The rats in the control group were injected with 200 uL of normal saline in an identical manner. The procedure was repeated every 48 h for a total of six doses. The DFO dose and injection schedule were based on previous review of the literature [16, 18, 20].

### Postoperative course

After the operation, the rats were allowed free motion and unrestricted access to food and water. All rats were monitored daily for signs of inflammation, lethargy, and for general health by an experienced animal care technician. Two hours after the surgery, injections of buprenorphine at 0.01 mg/kg (Schering-Plough, Belgium) were administered and continued at 0.005 mg/kg for 3 days. An X-ray examination was then performed to confirm that the implant was in a satisfactory predetermined position. We found no knee dislocation or subluxation, which demonstrated that the joint was stable. We also found there was no periprosthetic gap visible to the naked eye on radiographs, which indicates that the prosthesis and bone were in close contact (Fig. 2).

In this study, there were two observation time points after prosthesis implantation: 2 weeks and 3 months post-surgery. At 2 weeks after implantation, 32 rats were sacrificed. Euthanasia was performed by overdosing 16 rats (8 in the DFO group and 8 in the control group) with sodium thiopental at 90 mg/kg (Schering-Plough, Belgium). The proximal tibia was explanted, the bones were stripped of the surrounding soft tissue, and the prosthesis was removed. The bones were then fixed in cold 4% formaldehyde for 1 day for immunohistochemical examination. Another 16 rats (8 rats in each group) were euthanized in the same way. The bone tissue was cut into portions approximately 0.5 × 0.5 × 0.5 cm in size. The whole process was completed within 30 min. The samples were subsequently placed into sterile cryopreservation tubes, pre-cooled in liquid nitrogen for 5 min, and finally stored in a liquid nitrogen tank (− 80 °C) for polymerase chain reaction (PCR). Three months after the knee surgery, the remaining 48 rats were sacrificed in the same way. All tibias containing the stabilized prosthesis were stripped of the surrounding soft tissue and subjected to micro-computed tomography (micro-CT) analysis (16 rats, 8 in each group), histopathology (16 rats, 8 in each group) and biomechanical testing (16 rats, 8 in each group) as detailed below.

### Quantitative real-time reverse transcription PCR (rt-PCR)

The expression of VEGF and vascular endothelial cell marker CD31 were detected by rt-PCR. Total RNA was extracted using the TRIZOL protocol (Sigma-Aldrich, MO, USA), and then concentrated and purified according to the manufacturer’s instructions. RNA integrity was measured using agarose gel electrophoresis. A Prime-Script First-Strand cDNA synthesis kit (Takara, Dalian, China) was used for reverse transcription. A SYBR® premix Ex TaqTM kit (Takara) was used for PCR amplification. Real-time quantitative PCR was performed at 57 °C for 30 cycles in the Opticon continuous fluorescent detector using IQTM SYBR green supermix (BioRad, USA). Each group of samples was measured three times. 2 ^−△△CT^ was used to analyze the relative expression of each group of genes. Rat 18S ribosomal RNA was used as the internal reference. The primers used are set out in Table 1.

**Table 1:** Primers for real-time PCR analysis

Gene	Forward Primer	Reverse Primer
VEGF	AGAAAGCCCATGAAGTGGTGA	GCTGGCTTTGGTGAGGTTTG
CD31	TTGTGACCAGTCTCCGAAGC	TGGCTGTTGGTTTCCACACT
Rat-18sRNA	GTAACCCGTTGAACCCCATT	CCATCCAATCGGTAGTAGCG

### Immunohistochemistry

At the same time, immunohistochemistry was performed to assess the expression of HIF-1a,VEGF, and CD31, as well as to assess the angiogenic potential of the periprosthetic bone tissue. Briefly, the upper tibial bone tissue from which the prosthesis was removed was fixed, decalcified, embedded in paraffin, and sliced to a thickness of 5 mm. The sections were deparaffinized and washed with phosphate buffered saline (PBS). The slice was immersed in 3% hydrogen peroxide for 10 min to block endogenous peroxidase activity and then rinsed several times in PBS. After being blocked with 1% bovine serum albumin (BSA) solution at room temperature for 1 h, sections were treated with a primary antibody overnight at 4 °C and incubated with the biotinylated secondary antibody for 30 min. Negative control sections were incubated with PBS solution. The following specific antibodies were used: HIF-1a, HIF-2a (Upstate Biotechnology, Lake Placid, New York, USA), VEGF (Upstate Biotechnology), and CD31 (Santa Cruz Biotechnology, Dallas, Texas, USA). Next, the slides were visualized using a streptavidin-biotin staining technique that involves peroxidase labeling. The nuclei were counterstained with hematoxylin. The sections were observed under an optical microscope (Olympus BH-2, Japan) and photographed using a high performance computer-aided image analysis system (Nikon H600l, Japan). The yellow cells were considered antigen-positive. Then, image analysis software (Image Pro Plus 6.0 software) was used for quantitative analysis of the positive stained images. Ten random fields in the bone marrow cavities in each section were selected to quantify the positive staining with an accumulated optical density value. The ratio of the accumulated optical density value to the corresponding observation area was used as the final quantitative parameter and recorded as the mean optical density value (MOD). Eight sections for each protein from each group were analyzed. After immunohistochemical staining for CD31, the formation of microvessels could be observed by light microscopy. The observer was blind regarding the identity of the two groups.

### Micro-CT scan analysis

**Fig. 3:**
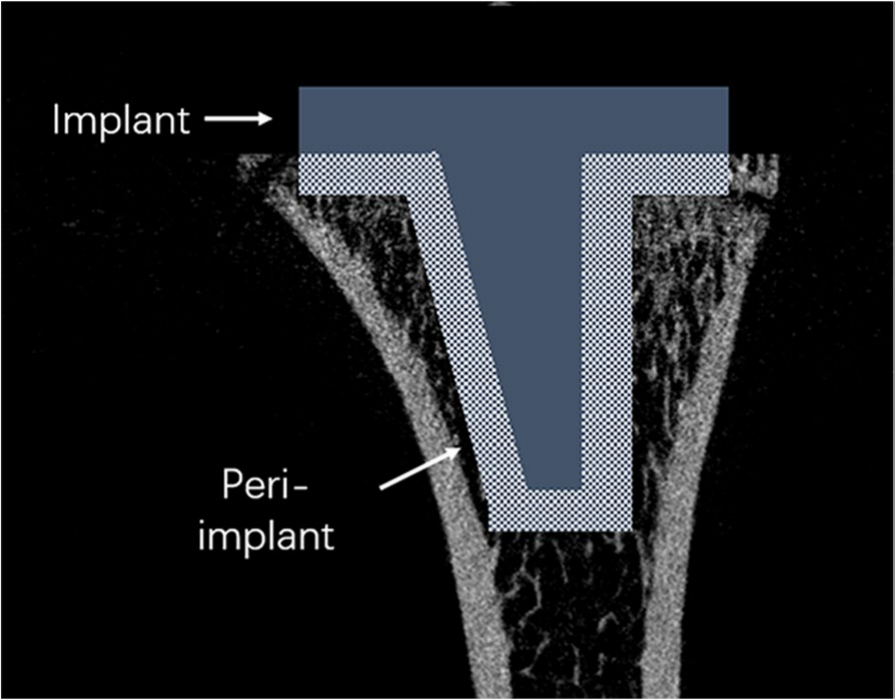
Volumes of interest for microCT measurements. At 3 months after the operation, peri-implant bone adjacent to the distal 2 mm of the implant was analyzed

Eight tibias in each group were harvested, the attached soft tissue was removed, and the gross specimens were examined. After the prosthesis was gently and manually removed, the bone around the prosthesis was examined by high-resolution micro-CT (Skyscan1176; Skyscan, Kontich, Belgium). The setting parameters for micro-CT were as follows: resolution ratio, 45 mm; volume, 50 kV; current, 500 UA. A 2-mm-thick layer of bone around the prosthesis was used as the volume of interest (VOI) (Fig. 3). The change in the amount of cancellous bone depends not only on the distance from the joint surface, but also on the distance from the cartilage growth zone. The implantation was a standardized operation. When we implanted the prosthesis, the stem passed through the growth plate. The distal end of the prosthesis was 6.5 mm under the joint surface and 4.5 mm under the growth plate. Continuous scanning was performed from the proximal to the distal tibia along the longitudinal axis of the tibia. The scanning length was 5 mm and the voxel size was 18 um. After 3D reconstruction of the trabecular bone was performed, the BMD, ratio of bone volume to tissue volume (BV/TV), trabecular thickness (Tb.Th), trabecular separation (Tb.Sp), and trabecular number (Tb.N) of the VOI were calculated with Mimics 19.0 software (Materialise, Leuven, Belgium).

### Biomechanics

Sixteen rats (8 in the DFO group and 8 in the control group) were sacrificed for biomechanical examination. Following euthanasia, eight tibias in each group were harvested, and the surrounding soft tissue was carefully removed. The proximal bone and prosthesis were clearly exposed. A WWD-10A electronic universal testing machine controlled by a microcomputer (Sanfeng Instrument Technology Co. Ltd., Changzhou, China) was used to pull out the prosthesis. The two ends of the tibia specimen and the prosthesis were fixed with self-curing plastic (methyl methacrylate) respectively. Two 1-mm diameter steel wires were set into the self-curing plastic at both ends. The steel wires were connected with clamps at both ends of the electronic universal testing machine to keep the longitudinal axis of the tibia perpendicular to the horizontal line of the ground. It was necessary to avoid axial and lateral stress to the prosthesis during the whole mechanical process. The load measurement accuracy of the tester was 0.01 N, and the loading speed was 0.1 mm/s. The pull-out test was performed to check the intensity of osseointegration. The stress-time curve was described by the self-contained software, and its peak value was recorded as the maximum pull-out force.

### Histopathology

Following euthanasia, eight retrieved upper tibial bones including implants in each group were processed for undecalcified histology. After fixation in 4% paraformaldehyde for 48 h, the implants were rinsed in water for 24-h, dehydrated in ethanol step by step, cleared in xylene for 12-h, and embedded in polymethyl methacrylate resin (Leica Microsystems GmbH, Wetzlar, Germany). After sufficient polymerization, each implant was cut along its vertical axis with a hard tissue microtome (SP1600 Leica Microsystems Concord, ON, Canada) to creat 7–8 sections. The vertical sections were in the coronal plane. The histological section was located 1.5 mm from the distal end of the prosthesis. The thickness of each slice was 70 um, and the three most central sections from each specimen were kept for polishing, staining with 1% toluidine blue, and histological analysis. A semi-automatic digital image analysis system was used for histomorphometric analysis of the bone morphology. The system includes a microscope (Olympus BX51, Olympus), digital camera (Nikon H600L) connected to the computer, and bone morphometry analysis software (Bioquant, USA). The bone-to-implant contact (BIC) value is the ratio of the length of direct contact between the surrounding bone tissue and the implant body to the total circumference of the implant body. The BIC value can be calculated by software to indicate the degree of bone integration [29–31].

### Statistical analysis

Before the investigation, the sample size was estimated by using the BIC as the primary variable. On the basis of our pilot studies, the standard deviation was assumed to be 13% in both the experimental and control groups, and an estimated difference of 20% was determined between the groups. A power calculation was performed with a confidence level of 95% (α = 0.05) and power (1-β) of 80%. This yielded an estimated sample size of seven knees per group. We used *n* = 8 for every examination at each time point to account for potential unexpected loss of animals during our experiments.

All the data are described as mean ± standard deviation. Student’s t-test was used for comparison of means between the two groups (SPSS version 20.0), and *p* < 0.05 was determined to be significant.

## Results

### Confirming the rat osteoporotic model

**Fig. 4:**
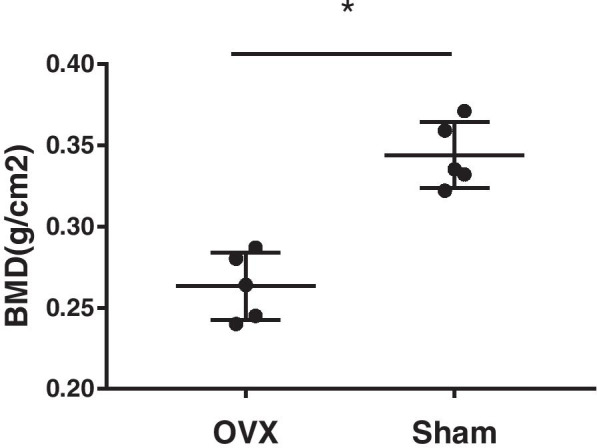
The bone mineral density (BMD) of trabecular bone in the OVX group was significantly lower than that of the sham group

Quantitative analysis by dual-energy X-ray absorptiometry showed that the BMD of the lumbar trabecular bone in OVX rats was 0.263 ± 0.021 mg/cm^2^, a decrease of 23.35%, and significantly lower than that of sham rats (0.343 ± 0.022 mg/cm^2^; *p* < 0.05; Fig. 4).

### Effect of DFO on angiogenesis

**Fig. 5:**
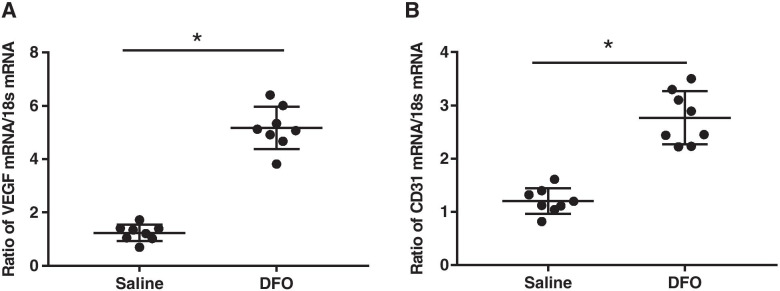
Deferoxamine (DFO) activated the HIF-1a signaling pathway under normoxic conditions, which could significantly increase the mRNA expression levels of VEGF, thereby further improving the mRNA expression levels of CD31. **A** The mRNA expression levels of VEGF in both groups. **B** The mRNA expression levels of CD31 in both groups. An asterisk (*) indicates *p* < 0.05

**Fig. 6.**
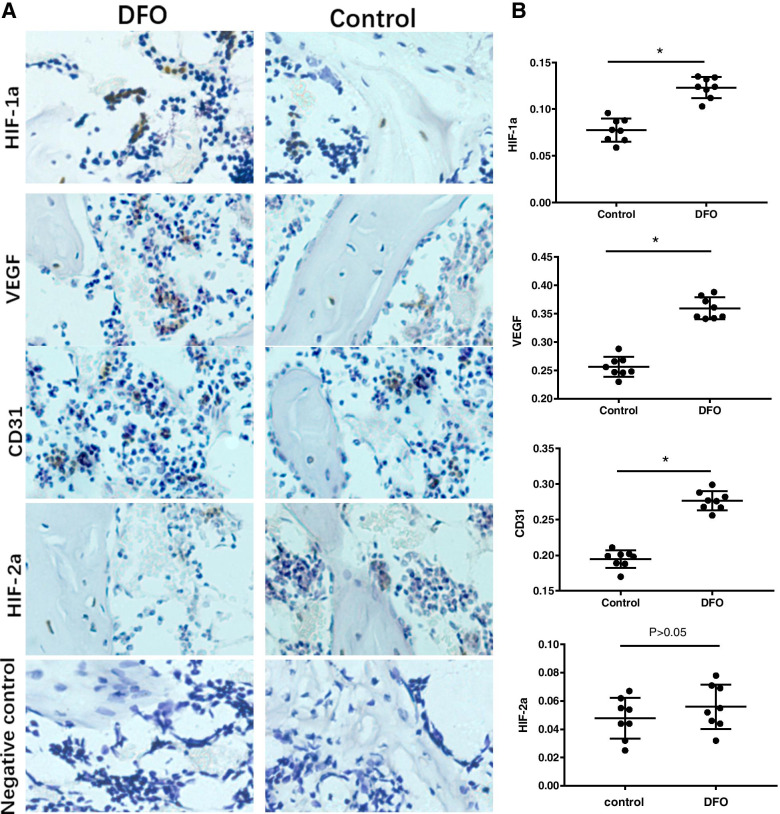
Sections of new bone tissue around the implants were examined by immunohistochemistry 2 weeks after surgery. **A** A representative photomicrograph showed there were more HIF-1a, VEGF, and CD31 positive cells in the DFO group than in the control group. The CD31 was expressed clearly in clusters, which indicated that there was more neovascularization. Only a small amount of HIF-2a was expressed in both groups. The appearance of a brown substance (black arrows) indicats antigen-positive staining (original magnification: 200 × per image, scale bars represent 20 μm). **B** Bar graphs showing the mean optical density of HIF-1a, VEGF, and CD31 in bone tissue. Semiquantitative analysis was performed for eight sections (10 fields per section) in each group, and the difference was obvious. There was no difference in HIF-2a expression between the two groups. An asterisk (*) indicates *p* < 0.05

Two weeks after the implants were placed, the mRNA levels of VEGF and CD31 were detected by real-time quantitative PCR. DFO significantly upregulated the mRNA expression levels of VEGF, which is an important angiogenic factor in the HIF-1a pathway [*p* < 0.05, Fig. 5(A)]. The comparision of the mRNA levels of CD31 in both groups showed that there was more significant angiogenesis in the DFO group than in the control group [*p* < 0.05, Fig. 5(B)]. The results of immunohistochemistry showed that there were significantly more positive granules of HIF-1a and VEGF in the sections of the DFO group than in those of the control group [Fig. 6(A)], and the mean optical density value was significantly higher in the DFO group than in the control group [*p* < 0.05, Fig. 6(B)]. We used the endothelial cell marker CD31 to monitor the formation of the vascular network and found that more CD31 positive cells formed tubular structures in the DFO group than in the control group [*p* < 0.05, Fig. 6(A)]. The expression levels of HIF-2a in both groups were also examined. However, there was no significant difference in the number of positive granulosa cells between the two groups. The mean optical density of the DFO group was slightly higher than that of the control group, but the difference was not statistically significant [*P* > 0.05, Fig. 6(B)].

### Effect of DFO on bone formation and integration

At 12 weeks after implantation, it could be seen that the osseointegration of the prosthesis was good, without loosening or displacement in the gross specimens [Fig. 2(H–I)]. Our benchmark for good osseointegration was that no displacement such as sinking, tilting, or rotation could be detected by the naked eye, and that the prosthesis could not be removed by clamping with forceps. However, in two cases in the control group, small cysts were found in the tibial plateau after the prosthesis was removed. After implantation, X-ray examination of the rat knee joint revealed no prosthesis loosening or displacement.

**Fig. 7:**
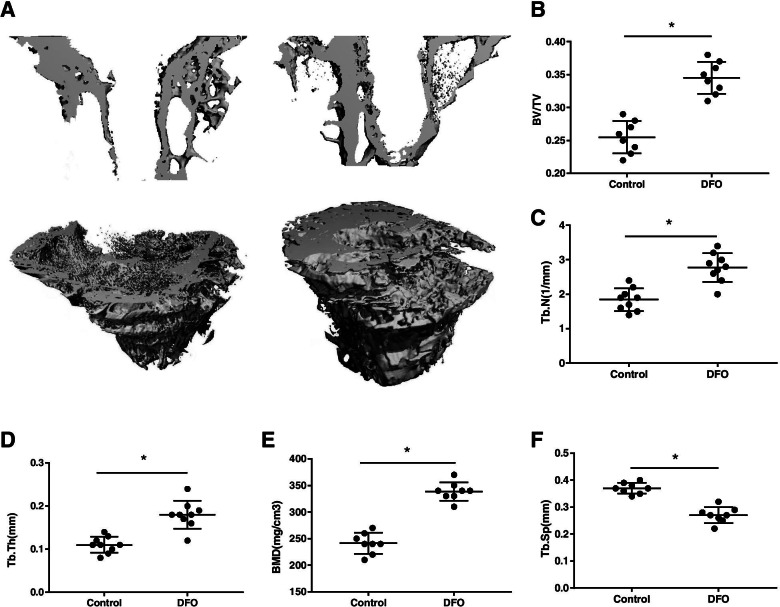
Bone formation around the prosthesis evaluated by micro-CT analysis. Twelve weeks after implant surgery, there were more new bone formation in the DFO group, I = empty space after the implant was withdrawn (**A**). BV/TV, Tb. N, Tb. Th and BMD in the DFO group over a uniform volume of interest were significantly higher than these measures in the control group, while Tb. Sp was much lower in the DFO group than in the control group (**B–F**). An asterisk (*) indicates *p* < 0.05

After the prosthesis was withdrawn, micro-CT was used to evaluate new bone formation around the prosthesis. The morphology of the new bone was quantitatively analyzed by micro-CT images, 3D reconstruction, and microstructure parameters. The micro-CT results showed that more bone tissue had formed around the prostheses in the DFO group, and the density and magnitude of bone trabeculae in the DFO group were higher than those in the control group. In the control group, the bone structure was sparse and loose, and the trabecular structure was disordered, which was not consistent with the direction of the main trabeculae. BV/TV, TB. N, TB. Th, and BMD for the DFO group were significantly higher than they were for control group, while TB.SP for the DFO group was significantly lower than it was for the control group [*p* < 0.05, *p* < 0.05, *p* < 0.05, *p* < 0.05, *p* < 0.05, respectively; Fig. 7(A–F)].

**Fig. 8:**
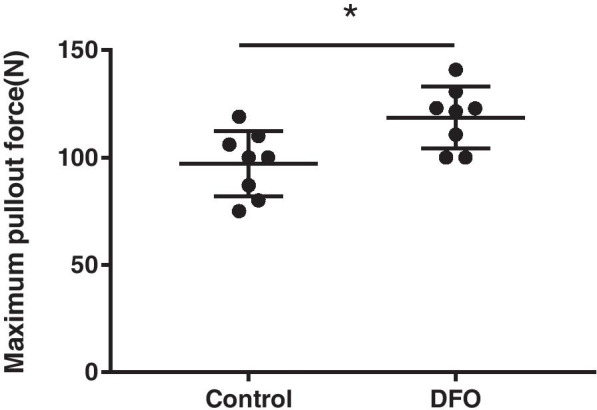
The biomechanical test results showed that the maximum pull-out force for the DFO group was significantly higher than it was for the control group. An asterisk (*) indicates *p* < 0.05

The biomechanical bond strength of the bone–implant interface was measured by the pull-out test. The maximum pull-out force (118.7 ± 14.4 N) for the DFO group was significantly higher than it was for the control group (97.1 ± 15.2 N), and the difference was statistically significant (*p* < 0.05; Fig. 8).

**Fig. 9:**
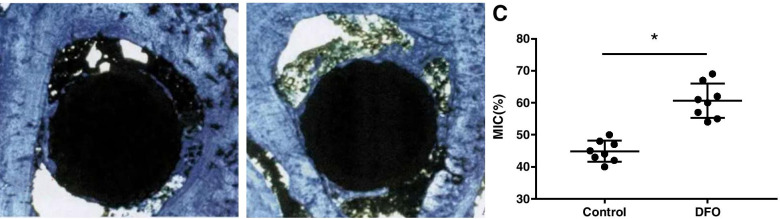
Results of the histopathological examination showed that the bone-to-implant contact (BIC) value in the DFO group was significantly higher than that in the control group (C). An asterisk (*) indicates *p* < 0.05

It can be seen from the undecalcified histological sections that there were more bone ingrowth into the prosthesis in the DFO group than in the control group. The MIC in the DFO group (60.6 ± 5.4%) was significantly higher than that in the control group (44.9 ± 3.3%), and the difference was statistically significant (*p* < 0.05; Fig. 9). In the DFO group, a small amount of soft tissue membrane surrounded the prosthesis; however, more soft tissue membrane was found in the control group.

## Discussion

In this study, we designed a knee prosthesis for rats and created a novel animal model of knee replacement surgery. By implanting the prosthesis into the osteoporotic proximal tibia, we found that local administration of the hypoxia simulant DFO to activate the HIF-1a signaling pathway could increase osteogenesis around the prosthesis in the osteoporotic bone, promote osseointegration, and improve the stability of the prosthesis. The results from micro-CT showed that the BV/TV, TB. N, TB. Th, and BMD of the bone trabeculae around the prosthesis were significantly increased, while Tb. Sp showed an obvious decrease, which indicates that the osteoporosis had improved. Biomechanical experimental results demonstrated that the binding force between the prosthesis and the surrounding bone increased significantly. Results obtained from histopathological analysis showed bone–implant integration also increased.

We successfully created an animal model of knee replacement surgery in osteoporotic rats through two sequential operations. The first operation involved an ovariectomy, which is a traditional and successful method used to model osteoporosis. This model can simulate the osteoporosis that occurs because of estrogen deficiency in older postmenopausal women [28]. This animal model of osteoporosis was used to more closely simulate the bone characteristics of older patients undergoing joint replacement. In the second operation, the tibial articular surface of the knee joint was replaced by cementless fixation to creat an animal model of hemiarthroplasty of the knee. This model differed from prior animal models of bone integration that used implanted titanium rods or screws outside or inside the joint [32–36]. Niels et al. [37] developed a knee replacement model that used an alloy prosthesis on the femur side and a high-density polyethylene prosthesis on the tibia side, both with cementless fixation; the prosthesis became loose and was displaced after the operation. Kenneth [38] used a polyetheretherketone material fixed with bone cement to make a successful rat model of tibial knee replacement. Carli et al. [39] made a mouse model of tibial knee replacement with a titanium prosthesis. Ours is the first study to use a titanium prosthesis in a knee replacement model in rats. Of course, the knee joints of humans and rats are different. The human knee bears weight in extension, while the rat knee bears weight in flexion. Additionally, there are differences in the range of motion and the angle of motion of the knee joint when walking. However, the main functions of the knee in rats are weight-bearing, stretching, and flexing. Our prosthesis can manage these functions. However, because of the lack of a stable device on the sagittal plane, the risk of forward dislocation of the knee joint is of concern. Fortunately, there was no evidence of joint dislocation in the postoperative physical and X-ray examinations. The theory of human joint stability may not apply to rats. The rats used the operatively treated leg normally postoperatively with no gait alterations after the immediate postoperative period. This observation suggests that the rats were bearing weight. In contrast to previous studies which investigated the non-weight-bearing state, we studied is that we are studying the osseointegration of animals in the weight-bearing state, which is closer to the situation in clinical practice. Although this is the first time we have used this kind of physiologically loaded intra-articular rat model, we have previously used a similar implant in the tibia of mice. This model is a viable platform on which to study pharmacologic enhancement of the bone–implant interface.

The dosage used in this experiment was determined with reference to the previous literature [40, 41]. This dose has produced obvious effects in many experimental situations, including distraction osteogenesis, fracture healing, and promoting osteointegration of artificial bone in repairing segment defects of long bones, and no serious side effects have been reported. For these reasons, we adopted this dose in our study. Further research is needed to determine the optimal dose. The time period of administration is the active period of tissue repair, during which time the drug plays the most active role. We adopted the method of intra-articular injection for local administration, which allows the drug to penetrate around the prosthesis and the bone to exert its pharmacological effects. Because of the narrow joint cavity of rats, only a small amount of saline (200 ul) was used for drug dissolution to prevent excessive drug extravasation. We chose 2 weeks and 3 months postoperatively as observation time points. At 2 weeks after the operation, blood vessels and scar tissue were actively forming, which is conducive to the detection of cytokines. At 3 months after surgery, the drug effect had disappeared, but mature bone had formed. We were particularly interested in the mature and stable bone tissue around the prosthesis, which could maintain the long-term stability of the implant.

DFO is a hypoxia simulator. It can inhibit the activity of prolyl hydroxyprolidase by combining with iron, thus causing the accumulation of HIF-1a in the nucleus. Through combining with the target gene, HIF-1a can stimulate the production of VEGF and promote the regeneration of blood vessels. Blood vessels transport not only oxygen and nutrition, but also transport growth factors and bone progenitor cells, all of which are necessary for the regeneration of bone. Through regulating the interaction among osteoblasts, osteoclasts, bone cells, and vascular cells, local bone growth is promoted [16, 21, 42–45]. Lowered levels of angiogenic factors such as VEGF and the resulting reduction in the rate of bone vessel formation are causes of poor bone repair. Therefore, the basic strategy for enhancing osteoporotic bone regeneration is to promote osteogenesis or angiogenesis. Drugs such as bisphosphonates can also be used to inhibit bone resorption [46]. Hypoxia is one of the main driving forces of angiogenesis–osteogenesis coupling. HIF is the key regulator of the coupling mechanism. Farberg et al. [23] conducted a study of distraction osteogenesis and found that injection of DFO into the distraction gap of bone enhanced the activity of all cells, including bone precursor cells and endothelial cells. As long as the DFO solution reaches the site, new bone formation can be clearly seen [47]. Studies have shown that local administration of DFO into fracture sites can promote fracture healing in long bones [16, 17]. Drager et al. [20] implanted microporous dicalcium phosphate dihydrodrate (brushite) grafts into the bone defects of rabbits and found that local injection of DFO significantly promoted bone integration. After topical administration of DFO carried by a polylactic acid scaffold to osteoporotic bone defects in rats, Jia et al. [25] found that vascular networks appeared denser around the DFO-containing scaffolds than they did around the untreated scaffoldsthis promoted new bone formation and repair of the bone defects. In our study, we found that local administration of DFO promoted the regeneration of blood vessels and bone around implants in osteoporotic bone, and ultimately promoted osseointegration.

PCR results showed that the mRNA levels of VEGF and CD31 were higher in the DFO group than in the control group, which demonstrated that abundant angiogenesis was involved in this osteogenesis process. This result was also confirmed by immunohistochemistry. We adopted an immunohistochemical method to detect the expression of HIF-1a and HIF-2a in the two groups, because DFO also stabilized the HIF-2a protein and further upregulated the expression of VEGF. The results showed only a small amount of HIF-2a expression in the two groups, with no difference between the groups. However, more HIF-1a protein was found in the DFO group, proving that DFO acts by stabilizing the expression of HIF-1a. Thus, in our study the bone formation around the prosthesis was promoted at least partially through the HIF-1a/VEGF pathway. Hypoxia is a powerful stimulus to angiogenesis. VEGF is the most powerful angiogenic factor in the downstream of the HIF-1a pathway [13]. It can promote the proliferation, migration, and tubular formation of endothelial cells by binding with specific receptors of vascular endothelial cells. In the immunohistochemistry experiment, the positive rate of CD31 in the experimental group significantly increased, and there were many clusters of positive cells, which demonstrated the existence of a large amount of neovascularization, while the expression level of CD31 in the control group was low. Rena et al. [48] used a biodegradable calcium phosphate complex to fill defects in a rat femoral defect model. After DFO was administered locally, the effect of angiogenesis was clearly enhanced. The number of blood vessels detected by angiography in the DFO group was twice that in the control group.

After implantation, we took X-ray of the rat knee joint and found no loosening or displacement of the prosthesis. We also detected no loosening in the gross specimens. The results of micro-CT indicated that compared with the control group, BMD, BV/TV, TB. N, and TB. Th in the DFO group increased significantly, while TB.SP decreased. These findings demonstrate that the bone formation around the prosthesis increased, and the osteoporosis improved. Jia et al. [25] implanted a polylactic acid scaffold with DFO into bone defects in the femurs of osteoporotic rats. One month after the operation, the micro-CT data showed that more bone had regenerated in the DFO implantation group, and that BV/TV, Tb. N, and Tb. Th were significantly higher in the DFO group than in the control group, which was consistent with the results of this study. Our findings suggest that enhancement of bone formation around the prosthesis will inevitably increase bone integration, thus increasing the long-term stability of the prosthesis. Zhou et al. [10] implanted prostheses in rat knees and concluded that the stability of the prosthesis was enhanced after the bone mass and bone density around the prostheses were increased, which is consistent with our conclusions. It is important to observe the stability of the implant and to monitor the gap between the bone and the implant, but it is difficult to detect the size of the gap using micro-CT because of the artifacts produced by the metallic implant. Therefore, we pulled the prosthesis out and detected the bone mass around the prosthesis to indirectly measure the amount of osseointegration. The more bone mass present around the prosthesis, the better is the osseointegration. Implant removal has an effect on the micro-CT and immunohistochemistry. When removing the implants, a small amount of bone tissue will be taken away due to osseointegration, which will reduce the bone mass around the prosthesis. The irregular contour of the inner bone surface caused by the loss of bone mass can be seen on CT images. Therefore, we removed the prosthesis very gently to prevent excessive bone loss.

The biomechanical results showed that the maximum pull-out force for the DFO group was significantly higher than it was for control group, which indicates that DFO can promote the strength of osseointegration of the prosthesis and improve its stability. The degree of bone integration can be seen more directly from the results of histopathological sections. Drager et al. [49] found that local administration of DFO can promote the integration of an artificial bone graft with host bone in animal experiments. Yu et al. [50] implanted a titanium prosthesis adhered by DFO into the bone of rats and observed that the bone growth around the prosthesis was significantly enhanced. Although there were no cases of loosened implants, we believe that if the observation time was prolonged, there would have been cases of implants loosening.

Our results show that using DFO to activate the HIF-1a signaling pathway can increase angiogenesis and further promote bone growth and integration with the implant. The mechanisms by which bone growth is promoted are complex. DFO can inhibit the differentiation and function of osteoclasts through the RANKL/OPG pathway, thus reducing the loss of bone mass. Additionally, DFO can also enhance the classic Wnt/ß-catenin signaling pathway in osteoblasts, stimulate bone marrow mesenchymal stem cells to differentiate into osteoblasts and strengthen osteoblast activity, and ultimately promote bone formation [50, 51]. Moreover, HIF-1a not only upregulates VEGF to promote the vasculogenic–osteogenic pathway but also directly regulates osteoblasts and promotes the proliferation, differentiation, and mineralization of osteoblasts. HIF-1a also enhances the Wnt/ß-catenin signaling pathway and regulates the expression of the target gene Runx2, which stimulates mesenchymal stem cells to differentiate into osteoblasts, with the involvement of a variety of cytokines. This is a relatively independent mechanism of VEGF [52, 53]. HIF-2a can also regulate angiogenesis and bone growth, but it mainly plays a role in chronic hypoxia [54]. Further exploration of these mechanisms will lead to a better understanding and the development of new treatments for promoting osteointegration.

The idea that osteoporotic patients can be treated with total knee arthroplasty has been widely accepted. Much attention has been focused on how to improve the success rate. The long-term success of orthopedic implants is largely dependent on the degree of osseointegration with the surrounding bone. The trabeculae of osteoporotic bone is sparse and thin, and the mechanical support force to the prosthesis is insufficient compared with normal bone, which can lead to cancellous bone bearing the construct collapse, resulting in prosthesis displacement and subsidence, and possibly causing periprosthetic fracture. Therefore, increasing the bone mass around the prosthesis is an important preventive measure.

In this study, no complications such as infection or necrosis were found in rats after administration of DFO. Additionally, DFO is cheap and widely used, which makes it an encouraging treatment prospect. Although our study focused on a knee replacement model, we speculate that the experimental results are also generalizable for hip replacement. There are two points to be improved upon in the future. First, the tibial prosthesis should be designed with a shape similar to a meniscus with a high perimeter, which is more conducive to the stability of the joint. Second, the systemic administration of DFO to promote bone integration should be investigated, given that some studies have shown that systemic administration of DFO may improve osteoporosis.

## Conclusions

This study suggests that the local administration of DFO to activate the HIF-1a signaling pathway can promote osteogenesis and osseointegration with a prosthesis in osteoporotic bone. DFO provides a potential therapeutic target and treatment scheme for osteoporotic bone integration.

## Data Availability

The datasets used and/or analysed during this study are available from the corresponding author on reasonable request.

## Abbreviations

DFO: Deferoxamine
PCR: Polymerase chain reaction
rt-PCR: Reverse transcription PCR
micro-CT: Micro-computed tomography
HIF-1a: Hypoxia inducible factor-1a
VEGF: Vascular endothelial growth factor
PBS: Phosphate buffered saline
BMD: Bone mineral density
BIC: Bone implant contact
MOD: Mean optical density
VOI: Volume of interest
BV/TV: Bone volume/tissue volume
Tb.Th: Trabecular thickness
Tb.Sp: Trabecular separation
Tb.N: Trabeculae number
OVX: Ovariectomy
